# *Adam21* is dispensable for reproductive processes in mice

**DOI:** 10.7717/peerj.12210

**Published:** 2021-09-23

**Authors:** Yinghong Chen, Chao Liu, Yongliang Shang, Liying Wang, Wei Li, Guoping Li

**Affiliations:** 1 State Key Laboratory of Stem Cell and Reproductive Biology, Institute of Zoology, Stem Cell and Regenerative Medicine Innovation Institute, Chinese Academy of Sciences, Beijing, China; 2 University of Chinese Academy of Sciences, Beijing, China; 3 Fertility Preservation Lab, Reproductive Medicine Center, Guangdong Second Provincial General Hospital, Guangzhou, China; 4 Advanced Medical Research Institute, Shandong University, Jinan, China; 5 The MOH Key Laboratory of Geriatrics, Beijing Hospital, National Center of Gerontology, Beijing, China

**Keywords:** *Adam21*, Spermatogenesis, Male infertility, ADAMs

## Abstract

**Background:**

As a group of membrane-anchored proteins, the proteins containing a disintegrin and metalloprotease domain (ADAMs) control many biological processes, especially for male fertility. Mouse* Adam21* was previously found to be specifically expressed in the somatic cells and germ cells of testes, but its functional role during spermatogenesis and male reproductive processes is still unknown.

**Methods:**

*Adam21*-null mice were created using the CRISPR/Cas9 system. Quantitative real-time PCR was used for analyzing of gene expression. Histological, cytological and immunofluorescence staining were performed to analyze the phenotypes of mouse testis and epididymis. Intracellular lipid droplets (LDs) were detected by Oil red O (ORO) staining and BODIPY staining. Fertility and sperm characteristics were also detected.

**Results:**

Here, we successfully generated an *Adam21* conventional knockout mouse model via CRISPR/Cas9 technology so that we can explore its potential role in male reproduction. We found that male mice lacking *Adam21* have normal fertility without any detectable defects in spermatogenesis or sperm motility. Histological analysis of the seminiferous epithelium showed no obvious spermatogenesis difference between *Adam21*-null and wild-type mice. Cytological analysis revealed no detectable defects in meiotic progression, neither Sertoli cells nor Leydig cells displayed any defect compared with that of the control mice. All these results suggest that *Adam21* might not be essential for male fertility in mice, and its potential function still needs further investigation.

## Introduction

About ten to fifteen percentage of couples are affected by infertile problems, with approximately equal contributions from both sides (Cooke & Saunders, 2002). Male infertility can be categorized as azoospermia (with zero sperm count), oligospermia (with diminished sperm count), Asthenozoospermia (with reduced motility of sperm), teratozoospermia (with abnormal sperm morphology), as well as combinations of these multiple defects (Huang et al., 2020b). Spermatogenesis is a complex and highly orchestrated process in the seminiferous epithelium where various germ cells undergo successive mitotic divisions, meiotic divisions, spermiogenesis and finally the mature spermatozoa (Hess & Renato De Franca, 2008; Wang et al., 2019). More than 2000 genes were reported to be expressed only in the testis (Schultz, Hamra & Garbers, 2003). Nevertheless, the functions of many this kind of genes remain to be elucidated.

A disintegrin and metalloproteinase (ADAMs) are a fascinating family of membrane-anchored, multidomain and multifunctional proteases. All ADAMs contain pro-metalloprotease, disintegrin, cysteine-rich, EGF-like, spacer, transmembrane, and cytoplasmic tail domains, and they regulate critical events that occur at the cell surface, including extracellular and intracellular signaling, cell adhesion, and cell migration (Weber & Saftig, 2012; White et al., 2005). ADAMs were initially found to be a novel type I transmembrane glycoproteins during an analysis of sperm-egg fusion (Blobel et al., 1992; Wolfsberg et al., 1993; Wolfsberg et al., 1995). Until now, about 40 ADAMs family members have been reported in the mammalian genome, including 21 members in humans and 37 members in mice, about half of these genes are expressed specifically or predominately in testis, which implies their potential function in male reproduction (Reiss & Saftig, 2009). The majority of the testis-specifically expressed ADAM proteins are produced in spermatogenic cells as precursors and processed by removing prodomains or both prodomains and metalloprotease domains to form mature and functional proteins, and these modified proteins are secreted and present on the surface of mature sperm (Cho, 2012). More importantly, some of these surface-displayed ADAMs in sperm can form complexes, such as ADAM1-ADAM2 (Cho et al., 2000), ADAM2-ADAM3-ADAM4 (Han et al., 2009), ADAM2-ADAM3-ADAM5 (Kim et al., 2006), and ADAM2-ADAM3-ADAM6 (Han et al., 2009), which are indispensable for sperm–egg interactions, and can help sperm migration from the uterus to the oviduct in mice. For example, *Adam1*^−/−^, *Adam2*^−/−^ and *Adam3*^−/−^ mice displayed fertilization defects. The depletion of *Adam1*, *Adam2 or Adam3* impaired both sperm migration from the uterus into the oviduct through the uterotubal junction (UTJ) and binding of sperm to zona pellucida (Cho et al., 1998; Nishimura et al., 2004; Xiong, Wang & Shen, 2019; Yamaguchi et al., 2009), Additionally, the knockout of *Adam6* in mice causes male subfertility and deficits in sperm ascent into the oviduct (Voronina et al., 2019). Currently, some of the reproductive ADAMs are well-studied, broadening our understanding of the molecular mechanisms underlying sperm functions and fertilization. But the function of some testis-specifically expressed ADAMs still need further investigation.

The *Adam21* gene was first cloned from a human testis cDNA library, and it mapped to human chromosome 14 and mouse chromosome 12 (Hooft van Huijsduijnen, 1998; Seldin, Hirohata & Apte, 2000). *Adam21* (also known as *Adam31*) mRNA was initially reported to be exclusively present in testes examined by Northern blot (Liu & Smith, 2000). *Adam21* was lately reported to produce two types of transcript isoforms with different developmental stages and cellular localizations by reverse transcription-polymerase chain reaction (RT-PCR) (Yi et al., 2010). ADAM21 protein was predominantly expressed in Leydig cells and Sertoli cells in the testes of mice (Liu & Smith, 2000). Moreover, ADAM21 protein was also found to be expressed in the TM3 Leydig cell line, TM4 Sertoli cell line and testicular germ cells but absent from mature sperm (Yi et al., 2010). TM3 and TM4 are two distinct epithelial cell lines derived from testis of the immature BALB/c mouse. These cell lines have been identified as Leydig (TM3) and Sertoli (TM4) cells (Mather, 1980). In addition, *Adam21* mRNA and ADAM21 protein were also found in the neurogenic subventricular zone (SVZ) compartment of adult rats and mice, and it is associated with neurogenesis and axonal growth in developing and adult rodent central nervous system (Yang, Baker & Hagg, 2005). All these previous results suggest that the testis-specifically expressed ADAM21 protein might participate in male reproduction. To test this possibility, we generated *Adam21* conventional knockout mice *via* CRISPR/Cas9 technology, but found that the knockout of *Adam21* didn’t display any reproductive defect in either male or female mice, indicating that this testis-specifically expressed gene is dispensable for mouse fertility.

## Materials & Methods

### Animal experiments

Mice (C57BL/6N) were obtained from the Experimental Animal Center of Institute of Zoology and maintained in a 12:12 light/ dark cycle with food and water available *ad libitum* in cages held at 23 ± 2 °C. All individualized ventilated cages were capable of hosting at most five mice, and mice were randomly divided into different cages. An *Adam21*-knockout mouse model (C57BL/6N) was created by CRISPR/Cas9-mediated genome engineering, and two guide RNAs (5′-GCCAGGACACAATCTCGACATGG-3′and 5′-AGCCGCCTATGCACTAAGTTTGG-3′) were designed for this study, which will be co-injected with Cas9 into fertilized eggs for knockout mice production. All the 2-month male mice were sacrificed by cervical dislocation before testes collection. All the experiments and studies on laboratory animals were carried out in accordance with guidelines approved by the Institutional Animal Care and Use Committee of the Institute of Zoology (IACUC-#08-133, IOZ20180013), Chinese Academy of Sciences.

### RNA extraction and quantitative real-time PCR

Total RNA was extracted from wild-type and *Adam21*-knockout adult mice testes or other tissues as previously described (Xu et al., 2016). cDNA was synthesized by the PrimeScriptTM RT Reagent Kit (TaKaRa, RR037A). Primer sets for *Adam21* and *β-actin* were used (Table S2). Real-time PCR was performed using the Roche Light Cycler^®^ 480 System, and the results were analyzed with the LightCycle480 SW 1.5.1.

### Fertility assay

The male fertility assessment experiments were performed as previously described (Han et al., 2020). Each 8-week-old *Adam21*^+/−^ and *Adam21*^−/−^ male mice was caged with 2 *Adam21*^+/−^females (7 or 8 weeks old). The female fertility tests were carried out as previously described (Sun et al., 2018). 7-week-old *Adam21*^+/−^ and *Adam21*^−/−^ female mice was housed with *Adam21*^+/−^males (8–9-week-old). Copulatory plugs were checked daily, and plugged females with visibly growing abdomen were separated into single cages for monitoring pregnancy.

### Sperm motility and sperm count assays

Sperm were released in phosphate-buffered saline (PBS, Gibco, C14190500BT) from the incisions of the cauda epididymis for 10 min at 37 °C. And then the swim-up suspension was used for the analysis of sperm motility with an Olympus BX51 microscope through a 20X phase objective (OLYMPUS, Japan). Viewing areas in each chamber were imaged using a CCD camera (Olympus). The samples were analyzed *via* computer-assisted semen analysis (CASA) using the Minitube Sperm Vision Digital Semen Evaluation System (12500/1300, Minitube Group, Tiefenbach, Germany). The incubated sperm solution was then diluted 1:10 and sperm number was counted with a hemocytometer.

### Immunofluorescence

Spermatocyte surface spreading was carried out using the drying-down technique previously described (Peters et al., 1997; Xu et al., 2016). The 10ml aliquots from the epididymal sperm sample were spread onto the surface of slides, and then the slides dried at room temperature. Frozen testes were cut into 5µm thick sections using a cryo-microtome (CM1950, Leica Biosystems) and then mounted on slides. The frozen sections were firstly fixed using 4% paraformaldehyde (PFA, P1110, Solarbio), and then the fixed sections as well as surface-spread spermatocytes and spermatozoa were washed with PBS for 3 times and blocked in 5% bovine serum albumin(BSA) with 0.1% Triton X-100 for at least 30 min, incubated overnight at 4 °C with the corresponding primary antibodies, followed by incubation with the secondary antibodies at 37 °C for 1 h. Moreover, the intracellular lipid droplets (LDs) were stained with 1 µg/ml BODIPY–PBS solution for 10 min at room temperature (RT). Finally, 4′,6-diamidino-2-phenylindole (DAPI) was used to stain the nuclei. The images were taken by a Zeiss LSM 880 microscope. Antibody information was listed in Table S3.

### ORO staining

Oil red O (ORO) staining was performed as reported previously (Khawar et al., 2021). Briefly, 5 µm thick frozen testes sections were cut and fixed using 4% PFA for 15 min followed by washing three times with 1X PBS. After rinsing, sections were incubated in 60% (vol/vol) isopropyl alcohol for 5 min and air-dried. Subsequently, the air-dried sections were stained for 15 min using 60% ORO solution. Next, to remove the background staining, the slides were rinsed using 70% ethanol for 5 s. Subsequently, the slides were rinsed with water and counterstained using Harris hematoxylin. Glycerol/ PBS (9:1) was used to mount tissues and then processed further.

### Serum hormone measurement

Blood collected from control and mutant mice was clotted for 1 h at room temperature and centrifuged at 1,000 g for 20 min. The serum LH, FSH and testosterone levels were measured with ELISA kits (Beijing Sinouk Institute of Biological Technology).

### Harvesting of tissues and histological analysis

Testes and cauda epididymis were dissected immediately following euthanization by cervical dislocation. The tissues were then fixed in Bouin’s fixative for at least 24 h, dehydrated and embedded in paraffin; sections (5 µm) were cut and collected on glass slides. Following deparaffinization, the slides were stained with Haematoxylin and Eosin (H&E) or stained with Periodic Acid Schiff (PAS)-hematoxylin for histological analysis.

### Phylogenetic analysis

The phylogenetic trees were constructed using MEGA X (Kumar et al., 2018) with the Neighbor-Joining (NJ) method (Saitou & Nei, 1987), the bootstrap test (Felsenstein, 1985), 1,000 replicates) and Jones-Taylor-Thornton (JTT) model (Jones, Taylor & Thornton, 1992). The motif analysis was performed with the MEME Suite (Bailey et al., 2015) and TBtools (Chen et al., 2020). And the genes of ADAM family with testis-enriched or testis-specific expression have been summarized and described according to previous papers (Cho, 2012; Edwards, Handsley & Pennington, 2008; Weber & Saftig, 2012; Yi et al., 2010).

### Statistical analysis

All results are presented as the mean ± SEM. The statistical significance of the differences between the mean values for the various genotypes was measured using a two-tailed unpaired Student’s *t*-tests implanted in GraphPad prism 9. The *P < 0.05, **P < 0.01, ***P < 0.001, and ****P < 0.0001 levels were considered significant. ns, not significant.

## Results

### Expression pattern of *Adam21* and the generation of *Adam21*- knockout mice

**Figure 1:**
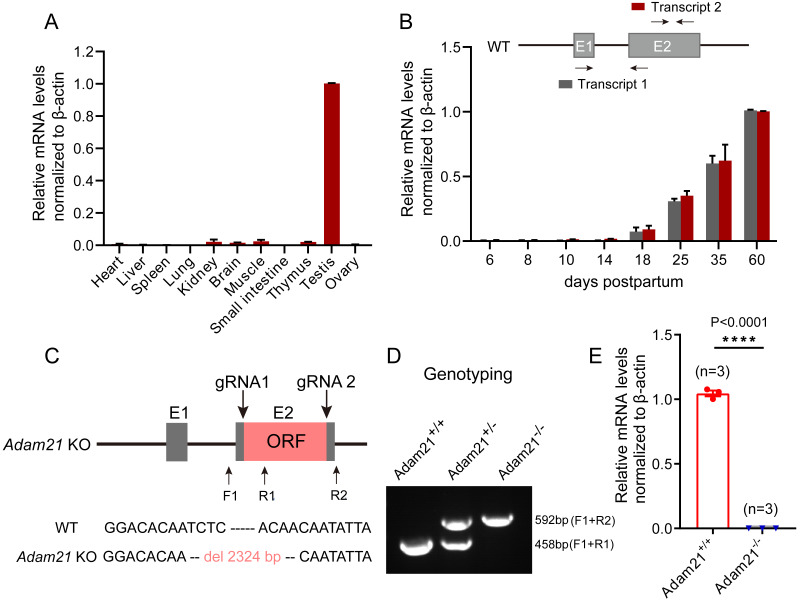
The expression of *Adam21* and the generation of *Adam21*^−/−^ mice. (A) Expression pattern analysis of *Adam21* in different tissues of 2-month-old adult mice by QRT-PCR. (B) Expression pattern analysis of *Adam21* during development of 6, 8, 10, 18, 25, 35, 60 dpp mice by QRT-PCR. (C) Schematic strategy of *Adam21*^−/−^ mice construction by CRISPR-Cas9-mediated genome editing. Sanger sequencing results of knockout mice to confirm the successful deletion of 2,324 bp in *Adam21* gene. (D) Genotyping of *Adam21*^−/−^ mice. Primer sets for genotyping were used (Table S1). (E) Quantification of *Adam21* mRNA level in testes from 2-month-old control and *Adam21*^−/−^ mice (*n* = 3 independent experiments). Data are presented as means ± SEM;^∗∗∗∗^*P* < 0.0001.

To gain insights into *Adam21* expression pattern, we firstly performed QRT-PCR analysis of *Adam21* in different adult tissues of mice or during development from juvenile to adult mice. After calculation and quantification, we found that *Adam21* prominently expressed in the testes (Fig. 1A). We noticed that *Adam21* expression was at minimal or background levels during the first wave of spermatogenesis at 18 days postpartum (dpp) (Fig. 1B), when the first wave of meiosis is about to be completed. These results are consistent with previous reports (Liu & Smith, 2000; Yi et al., 2010) and published transcriptome data from the Mouse ENCODE Project (Yue et al., 2014). The testis-specific expression of *Adam21* makes it a candidate gene to be involved in male fertility. To test this hypothesis, we generated *Adam21*-knockout mice *via* the CRISPR-Cas9 system that targeted the whole open reading frame (ORF) which is located at exon 2 of *Adam21* gene (Fig. 1C). Sanger sequencing and genotyping were performed to show that we have got mice with 2,324 bp deletion in exon 2 of *Adam21* (Figs. 1C and 1D). Furthermore, we found that the *Adam21* mRNA was completely absent in the *Adam21*^−/−^ testes compared with that of the *Adam21*^+/+^ testes by QRT-PCR (Fig. 1E), indicating that the knockout mice were indeed *Adam21*–null.

### *Adam21*-knockout mice have normal testis size and seminiferous tubules

**Figure 2:**
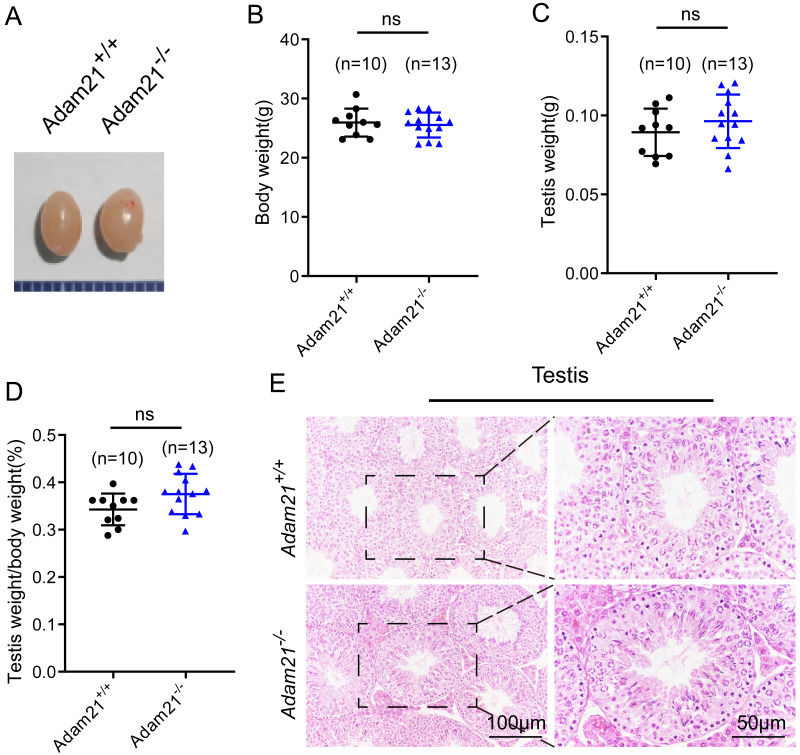
The knockout of *Adam21* doesn’t affect testis size and seminiferous tubule. (A) Representative image of testes from 2-month-old *Adam21*^+/+^ and *Adam21*^−/−^ mice. (B) Body weight of 2-month-old *Adam21*^+/+^ and *Adam21*^−/−^ male mice. (C) Testis weight of 2-month-old *Adam21*^+/+^ and *Adam21*^−/−^ mice. (D) Testis/body weight ratios in 2-month-old *Adam21*^+/+^ and *Adam21*^−/−^ mice. (E) Histological analysis of the seminiferous tubules of *Adam21*^+/+^ and *Adam21*^−/−^ mice by H&E staining. n: indicates independent experiments. Data are presented as means ± SEM; ns: indicates no difference.

*Adam21*^−/−^ mice were viable and appeared normal, displaying no obvious abnormalities in development or behavior. We examined the testis morphology as well as testis/body weight ratio of adult *Adam21*^−/−^ and *Adam21*^+/+^ mice, and no significant difference in testis size (Fig. 2A), body weight (Fig. 2B), testis weight (Fig. 2C), or the percentage of testis weight compared to body weight (Fig. 2D) between knockout and control male mice were noted. To examine more subtle testicular defects, we carried out histological examination of testes sections by hematoxylin-eosin staining, and the results revealed that seminiferous tubules of *Adam21*^−/−^ mice displayed normal structure and no obvious defects compared with that of wild-type mice were found (Fig. 2E).

**Figure 3:**
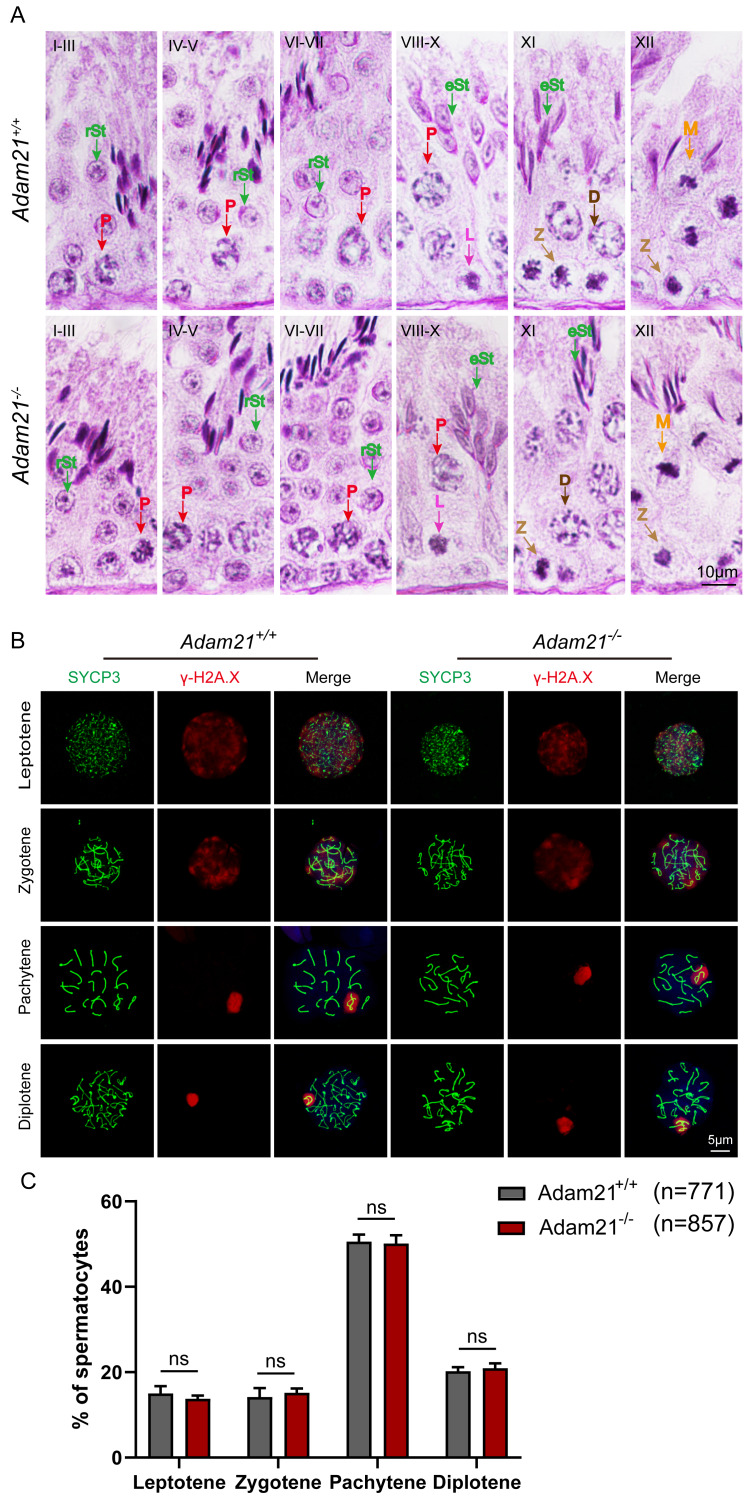
*Adam21*^−/−^ mice have normal spermatogenic and meiotic processes. (A) The PAS and hematoxylin staining was performed in *Adam21*^+/+^ and *Adam21*^−/−^ seminiferous tubules. L: leptotene spermatocyte, Z: zygotene spermatocyte, P: pachytene spermatocyte, M: meiotic spermatocyte, D: diplotene spermatocyte, rSt: round spermatid, eSt: elongating spermatid. (B) Immunofluorescence staining with antibodies against SYCP3 (green) and γ-H2AX (red) in *Adam21*^+/+^ and *Adam21*^−/−^ surface-spreading spermatocytes. (C) Meiotic stage frequencies in *Adam21*^+/+^ and *Adam21*^−/−^ testes. Data are presented as means ± SEM for 3 mice per genotype; ns: indicates no difference, the total number of spermatocytes counted is indicated (n).

### The knockout of *Adam21* doesn’t impair spermatogenesis and meiosis

Spermatogenesis is a cyclic process during which germ cells undergo a series of developmental steps following a tightly regulated time schedule. The cycle of the seminiferous epithelium can be subdivided into 12 stages in mouse testes according to steps in spermatid development using Bouin’s-fixed normal testes and sections stained with the Periodic Acid Schiff (PAS) technique and hematoxylin (Ahmed & De Rooij, 2009). To further explore the distribution and arrangement of spermatogenic cells in the seminiferous epithelium of *Adam21*^−/−^ mice, we carefully checked the 12 stages of spermatogenesis in *Adam21-* knockout testes. We found that the testicular tubules of knockout mice had well-organized architecture and normal spermatogenesis with presence of full array of germ cells ranging from spermatogonia to elongated spermatids which are similar to that of the wild-type testes (Fig. 3A). In order to analyze *Adam21* deficient spermatocytes, we identified the various stages of meiotic prophase by staining for a component of the synaptonemal complex, SYCP3 (Zickler & Kleckner, 1999) and a marker of the formation and repair of meiotic DNA double-strand breaks (DSBs), γ-H2AX (Hunter et al., 2001) in the surface-spread spermatocyte nuclei. We found that cells in meiotic prophase of *Adam21-*null mice have typical four different cytological stages: leptotene, zygotene, pachytene and diplotene meiotic cells (Fig. 3B). And then, we counted the number of cells at different stages of meiotic prophase and quantified the ratios of each meiotic prophase stage identified in the spermatocytes of *Adam21-* knockout and wild-type testes and found no obvious difference (Fig. 3C). Thus, we concluded that the knockout of *Adam21* doesn’t affect meiosis and spermatogenesis in mice.

### The knockout of *Adam21* doesn’t affect sperm counts, morphology and motility

**Figure 4:**
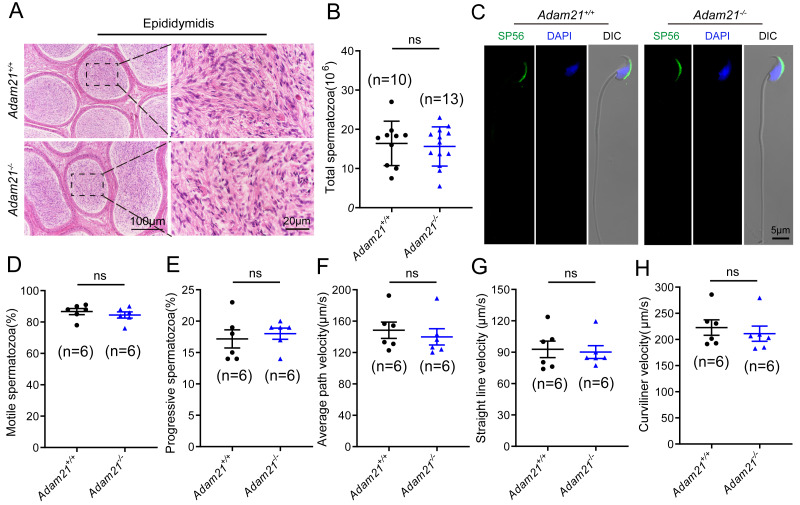
The sperm counts, sperm motility and sperm morphology of *Adam21*^−/−^ mice are normal. (A) Histological analysis of the caudal epididymidis of *Adam21*^+/+^ and *Adam21*^−/−^ mice by H&E staining. (B) The sperm counts in the caudal epididymidis were detected in *Adam21*^+/+^ and *Adam21*^−/−^ mice. (C) Immunofluorescence staining of sp56 (green) in *Adam21*^+/+^and *Adam21*^−/−^ spermatozoa. (D) Motile sperm in *Adam21*^+/+^ and *Adam21*^−/−^ mice. (E) Progressive sperm in *Adam21*^+/+^ and *Adam21*^−/−^ mice. (F) The average path velocity of sperm from *Adam21*^+/+^ and *Adam21*^−/−^ mice. (G) The straight line velocity of sperm from *Adam21*^+/+^ and *Adam21*^−/−^ mice.(H) The curvilinear velocity of sperm from *Adam21*^+/+^ and *Adam21*^−/−^ mice. n: indicates independent experiments. Data are presented as means ± SEM; ns: indicates no difference.

As sufficient sperm counts, vigorous sperm motility and well-defined sperm morphology are critical factors required for normal oocyte fertilization and overall pregnancy rate in mammals, and defects in any of these factors often cause male infertility. To investigate whether the absence of *Adam21* impairs spermiogenesis, we examined the cauda epididymis by histological analysis and found that sperm density of *Adam21*^−/−^ mice appeared to be normal in the cauda epididymis compared to that of *Adam21*^+/+^ mice (Fig. 4A). We further counted the total spermatozoa in the cauda epididymis and found that *Adam21*^−/−^ male mice had normal epididymal sperm numbers compared with that of their littermates (Fig. 4B). To further test whether the knockout of *Adam21* had any impact on sperm morphology, we performed single-sperm immunofluorescence using the acrosome-specific marker sp56, and DAPI was co-stained to indicate the sperm nucleus. The sperm morphological characteristics were observed using a confocal microscopy, and the results showed that the majority of knockout and wild-type sperm had normal morphology (Fig. 4C). In addition, we measured the motile sperm rate in *Adam21*^+/+^ and *Adam21*^−/−^ mice *via* CASA system, and the *Adam21*-depleted sperm displayed similar motility with wild-type sperm (Fig. 4D). And other motility-related parameters that we detected also showed no defects in *Adam21*^−/−^ mice compared with that of *Adam21*^+/+^ mice (Figs. 4E–4H), including the percentage of progressive spermatozoa, average path velocity (VAP), straight line velocity (VSL) and curvilinear velocity (VCL). In a nutshell, the *Adam21*-null mice showed no gross defects in sperm counts, sperm morphology and sperm motility.

**Figure 5:**
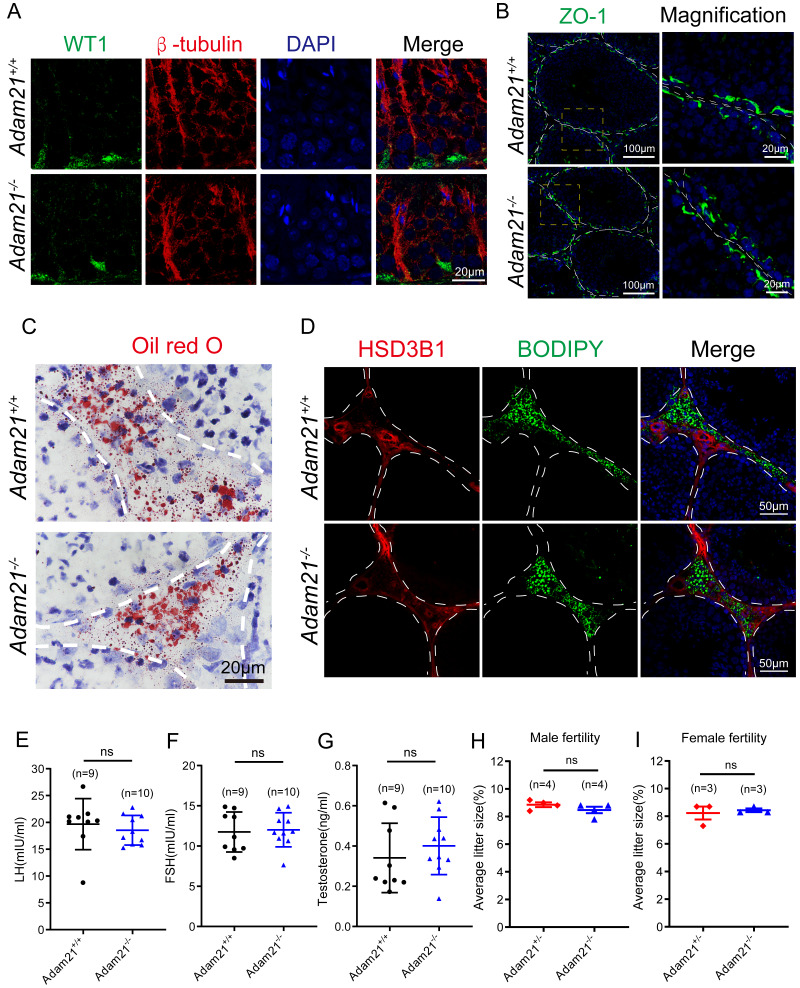
The knockout of *Adam21* doesn’t affect serum hormones and their fertility. (A) Immunofluorescence analysis using antibodies against WT1 (green) and *β*-tubulin (red) was performed in the seminiferous tubules of *Adam21*^+/+^ and *Adam21*^−/−^ mice. (B) Immunofluorescence staining of ZO-1 (green) on the testicular sections of *Adam21*^+/+^ and *Adam21*^−/−^ mice. (C) Oil red O staining performed on the testicular sections of *Adam21*^+/+^ and *Adam21*^−/−^ mice. (D) BODIPY staining (green) and immunofluorescence staining of HSD3B1 (red) on the testicular sections of *Adam21*^+/+^ and *Adam21*^−/−^ mice. (E) LH levels had no significant difference. (F) FSH levels had no significant difference. (G) Testosterone levels had no significant difference. (H) The average litter size of 2-month-old *Adam21*^+/−^ and *Adam21*^−/−^ male mice. (I) The average litter size of 2-month-old *Adam21*^+/−^ and *Adam21*^−/−^ female mice. n: indicates independent experiments. Data are presented as means ± SEM; ns: indicates no difference.

### The knockout of *Adam21* doesn’t affect luteinizing hormone (LH), follicle stimulating hormone (FSH), testosterone levels and their fertility

Spermatogenesis is regulated by both endocrine and paracrine hormones. LH and FSH secreted by the pituitary gland are main endocrine factors which interact with specific receptors (LHR and FSHR) expressed by the Leydig and the Sertoli cells, respectively. Testosterone synthesized by the Leydig cells mainly engages in the paracrine regulation of spermatogenesis (Chen, Ge & Zirkin, 2009; Cooke & Saunders, 2002). Since *Adam21* was expressed in the Sertoli cells and Leydig cells, we speculated that *Adam21* might have some relationship with the blood-testis barrier (BTB) integrity or hormonal interactions. We firstly analyzed the cytoskeletal structures and nuclei of Sertoli cells in *Adam21*^−/−^ and *Adam21*^+/+^ mouse testes by immunofluorescence staining with antibodies against *β*-tubulin and WT1, respectively (Liu et al., 2016; O’Donnell & O’Bryan, 2014). In *Adam21*^−/−^ testes, the microtubules were oriented in linear arrays parallel to the long axis of the Sertoli cells, from the base to the apex, forming a longitudinally oriented cage-like structure around the Sertoli cell nuclei, which were similar to the cytoskeleton and nuclei of Sertoli cells in that of the control testes (Fig. 5A). All these results suggested that the knockout of *Adam21* may not affect the morphology of Sertoli cells. We then detected the localization of ZO-1 which is a major tight junction (TJ) structural protein by immunofluorescence, but the result showed that the knockout of *Adam21* didn’t compromise the BTB integrity (Fig. 5B). We also detected the protein level of HSD3B1 which is a key steroidogenic enzyme by immunofluorescence and LDs stored in Leydig cells by ORO Staining (Fig. 5C) and BODIPY staining (Fig. 5D), while the results in the knockout mice displayed similarity to that of the control mice. Furthermore, we collected the serum of 2-month-old control and *Adam21* knockout mice, and then measured their LH (Fig. 5E), FSH (Fig. 5F) and testosterone (Fig. 5G) concentrations in the prepared sera, but we found that the levels of the three hormones in the *Adam21*^−/−^ mice were similar to that of the control, too. Therefore, the knockout of *Adam21* didn’t have adverse effects on the LH, FSH and testosterone concentrations in serum. To investigate whether *Adam21*^−/−^ mice were fertile, we conducted a fertility test of *Adam21*^−/−^ mice and *Adam21*^+/−^ mice, and found that neither male nor female *Adam21*^−/−^ mice displayed any infertility, they produce similar amount of litter to that of the control mice (Figs. 5H and 5I). Thus, we concluded that *Adam21* might be dispensable for reproduction in mice.

### Phylogenetic analysis of ADAMs family proteins

**Figure 6:**
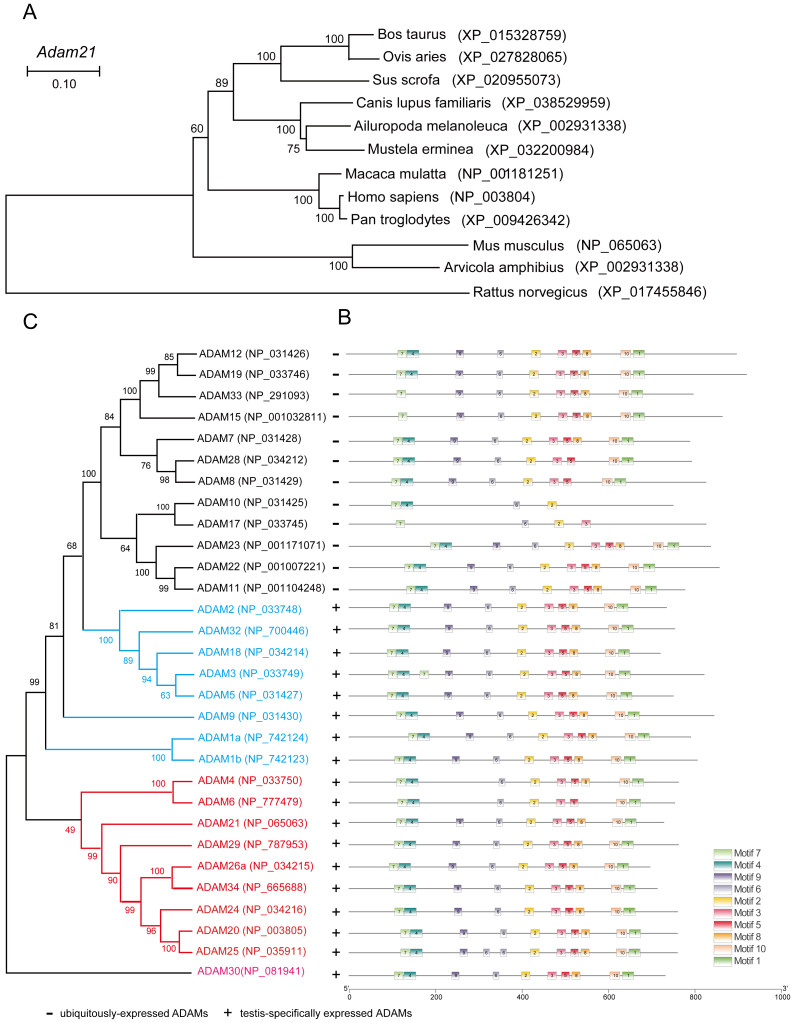
Phylogenetic relationships and conserved motif analysis of ADAMs. (A) Phylogenetic tree of the *Adam21* homologous proteins in different eutherian species. (B) The motif patterns of murine ADAMs were analyzed using the MEME Suite, and the 10 distinct MEME-motifs were displayed in different colored boxes. (C) Phylogenetic clustering by MEGA X software.

To address why the testis specific expressed gene is not essential to male reproduction, we conducted phylogenetic analysis of the ADAM21 related proteins. We found that ADAM21 is evolutionary conserved from mouse to human in mammals (Fig. 6A). In mice, the testis-specifically expressed ADAMs can be divided into two major groups according to gene structure. Group 1 contains ADAM1, 4, 6, 20, 21, 24, 25, 26, 29, 30 and 34, all of which lack introns in their open reading frames (ORFs). Group 2 includes ADAM2, 3, 5, 18 and 32, and all of them consist of multiple small exons and occupy large regions of the corresponding genome (Edwards, Handsley & Pennington, 2008; Weber & Saftig, 2012; Yi et al., 2010). The reproductive ADAMs contains the same domains reported in other ubiquitously-expressed ADAMs (Cho, 2012; White et al., 2005). To further assess the functional diversification of murine ADAMs, 10 conserved motifs were predicted using the MEME Suite (Fig. 6B). Additionally, ADAM21, 29, 26, 34, 24, 20 and 25 were clustered into a group in which bootstrap values show up 99% of the time using MEGA X software (Fig. 6C), suggesting these proteins might be redundant in their functions to make sure the genetics information could be transferred to the next generation efficiently.

## Discussion

Animal models have advanced our understanding of the reproductive development defects underlying human infertility. Studies on knockout mouse models have expanded our knowledge about the roles of specific genes engaged in stem-cell renewal, meiosis and spermiogenesis (Cooke & Saunders, 2002). Here, we focused on *Adam21*, a conserved and testis-specific gene, to study its role in reproduction. We found that loss of *Adam21* in male mice doesn’t show adverse impact on spermatogenesis and fertility. The architecture of testicular tubules, the progression of meiosis and sperm characteristics in *Adam21*^−/−^ mice are totally normal in comparison with that of the control mice. Additionally, all the testicular ADAMs in mice are expressed in spermatogenic cells. Apart from other ADAMs, *Adam21* is expressed in both testicular somatic and germ cells (Liu & Smith, 2000; Yi et al., 2010). And we didn’t find any defects in either Sertoli or Leydig cells because *Adam21*^−/−^ mice displayed no apparent changes in the protein level or distribution of ZO-1, WT1, *β*-tubulin and HSD3B1, and the intracellular lipid droplets (LDs) are also similar to that of the control mice. Furthermore, the reproductive related hormones such as LH, FSH and testosterone were not affected by *Adam21* knockout, suggesting that the ablation of *Adam21* didn’t affect the normal functions of Sertoli cells and Leydig cells.

Although *Adam21* was identified as an evolutionary conserved and testis-specific expressed gene, we didn’t find any developmental or reproductive defects after carefully examining the spermatogenesis and fertility of *Adam21-*null mice. According to our phylogenetic analysis, ADAM21 was divided into the same group with other 6 homologues in mice (ADAM29, 26, 34, 24, 20 and 25), and some of them or even all of them might have redundant functions in male reproduction. Among the 6 homologues, ADAM24 is essential for male fertility, and the knockout of *Adam24* leads to male subfertility in mice (Zhu et al., 2009), while others haven’t been carefully examined in mice. Thus, only all these redundant genes were knocked out, their potential functions can be revealed. In addition to ADAM21, mice lacking testis-specific ADAM32 were reported to have normal fertility, testicular integrity, and sperm characteristics (Lee, Hong & Cho, 2020), suggesting that *Adam21* may be not the only ADAM family members which is dispensable for fertility. The functional redundancy and compensation are well-documented in male fertility. Many family genes were reported to be dispensable for male mouse fertility, such as *Prss44, Prss46, and Prss54* (Holcomb et al., 2020); *Prss55* (Khan et al., 2018); *Tex37* (Khan et al., 2018) and *Tex55* (Jamin et al., 2021); *Rybp* (He et al., 2020); *Stk31/Tdrd8* (Zhou et al., 2014). The functional redundancy provides multiple ways to produce functional sperm, thus make sure their genetic information could be efficiently transferred into the next generation.

*Adam21* may not be required for normal spermatogenesis and reproduction. Alternatively, it may only be required for male reproduction during some stress conditions. For example, some genes have been reported to protect the spermatogenesis from toxic environmental stress, such as MAGE genes and *Ggnbp1,* they are not required for normal spermatogenesis, but can ensure proper gamete production in response to stress, while their knockout resulted in sensitiveness to genotoxic stress (Fon Tacer et al., 2019; Han et al., 2020). In addition to that, some chemicals and heavy metal have been reported to be toxic for the spermatogenesis, such as BPA, phthalates, dioxins, cadmium and so on (Bucci & Meistrich, 1987; Chung et al., 2011; Harman & Richburg, 2014; Zhang et al., 2013). Thus, although the knockout of *Adam21* does not affect the male reproduction in mice, it may make the knockout mice sensitive to these kinds of stresses. If this possibility could be demonstrated in mice, it may be applied to human beings because a lot of people’s sperm qualities are sharply decreased in the past decades due to either life-style changing or environmental pollutions (Deng et al., 2016; Huang et al., 2020a).

## Conclusions

Although *Adam21* was highly expressed in both testicular somatic and germ cells, the knockout of *Adam21* displayed neither testicular somatic cells nor germ cells defect in comparison to that of the control mice, and the knockout of this gene does not affect the reproductive processes at all in mice.

##  Supplemental Information

10.7717/peerj.12210/supp-1 Supplemental Information 1 Raw data of fertility test

10.7717/peerj.12210/supp-2 Supplemental Information 2 Raw data of hormone

10.7717/peerj.12210/supp-3 Supplemental Information 3 Supplementary Tables

10.7717/peerj.12210/supp-4 Supplemental Information 4 Raw data of RT-qPCR

10.7717/peerj.12210/supp-5 Supplemental Information 5 Raw data of sperm mobility

10.7717/peerj.12210/supp-6 Supplemental Information 6 Raw data of sperm count

10.7717/peerj.12210/supp-7 Supplemental Information 7 Raw data of body or testis weight

10.7717/peerj.12210/supp-8 Supplemental Information 8 ARRIVE Checklist

10.7717/peerj.12210/supp-9 Supplemental Information 9 Full-length uncropped blots of Adam21 genotyping results for Fig. 1

## Additional Information and Declarations

### Data Availability

The following information was supplied regarding data availability: The data are available as Supplementary Files.
