# Supplementary material for: Adam21 is dispensable for reproductive processes in mice
Source: PeerJ. 2021 Sep 23;9:e12210. doi: 10.7717/peerj.12210 (PMC8465997; doi:10.7717/peerj.12210)
Supplement: Supplemental Information 3 [file peerj-09-12210-s003.docx]

Table S1. PCR-related primer list.

| Gene | Primer sequence |
| --- | --- |
| ADAM21-F1: | 5’-CCTTTTATTATGGATGTCCTC-3’ |
| ADAM21-R1: | 5’-GAGGTCAATAAGGGATGGAAGTG-3’ |
| ADAM21-R2: | 5’-TGGCAAAGATAATAGACAAGG-3’ |

Table S2. Real-time PCR-related primer list.

| Gene | Primer sequence |
| --- | --- |
| ADAM21-transcript1-F | 5’-GGGCTTGATTTCTTCGTTCGG-3’ |
| ADAM21-transcript1-R | 5’-GGTCAATAAGGGATGGAAGTGCC-3’ |
| ADAM21-transcript2-F | 5’-CTCCCAATGGAAAACATACATCAG-3’ |
| ADAM21-transcript2-R | 5’-TGACACAACCACTTTTCCCAC-3’ |
| β-actin-F | 5’-GGTGGGAATGGGTCAGAAGG-3’ |
| β-actin-R | 5’-GTACATGGCTGGGGTGTTGA-3’ |

Table S3. Antibody information in this article.

| Antibody | Source | Dilution | Source | Reference |
| --- | --- | --- | --- | --- |
| sp56 | Mouse | 1:200 | QED Bioscience | 55101 |
| SYCP3 | Rabbit | 1:400 | Abcam | ab15093 |
| γH2A.X | Mouse | 1:400 | Millipore | 05-636 |
| HSD3B1 | Mouse | 1:200 | Santa Cruze | SC-515120 |
| ZO-1 | Rabbit | 1:200 | Invitrogen | 40-2200 |
| WT1 | Rabbit | 1:200 | Epitomics | S0268 |
| β-tubulin | Mouse | 1:200 | Abmart | M20005L |
