# Supplementary figures and images for: Adam21 is dispensable for reproductive processes in mice
Source: PeerJ. 2021 Sep 23;9:e12210. doi: 10.7717/peerj.12210 (PMC8465997; doi:10.7717/peerj.12210)

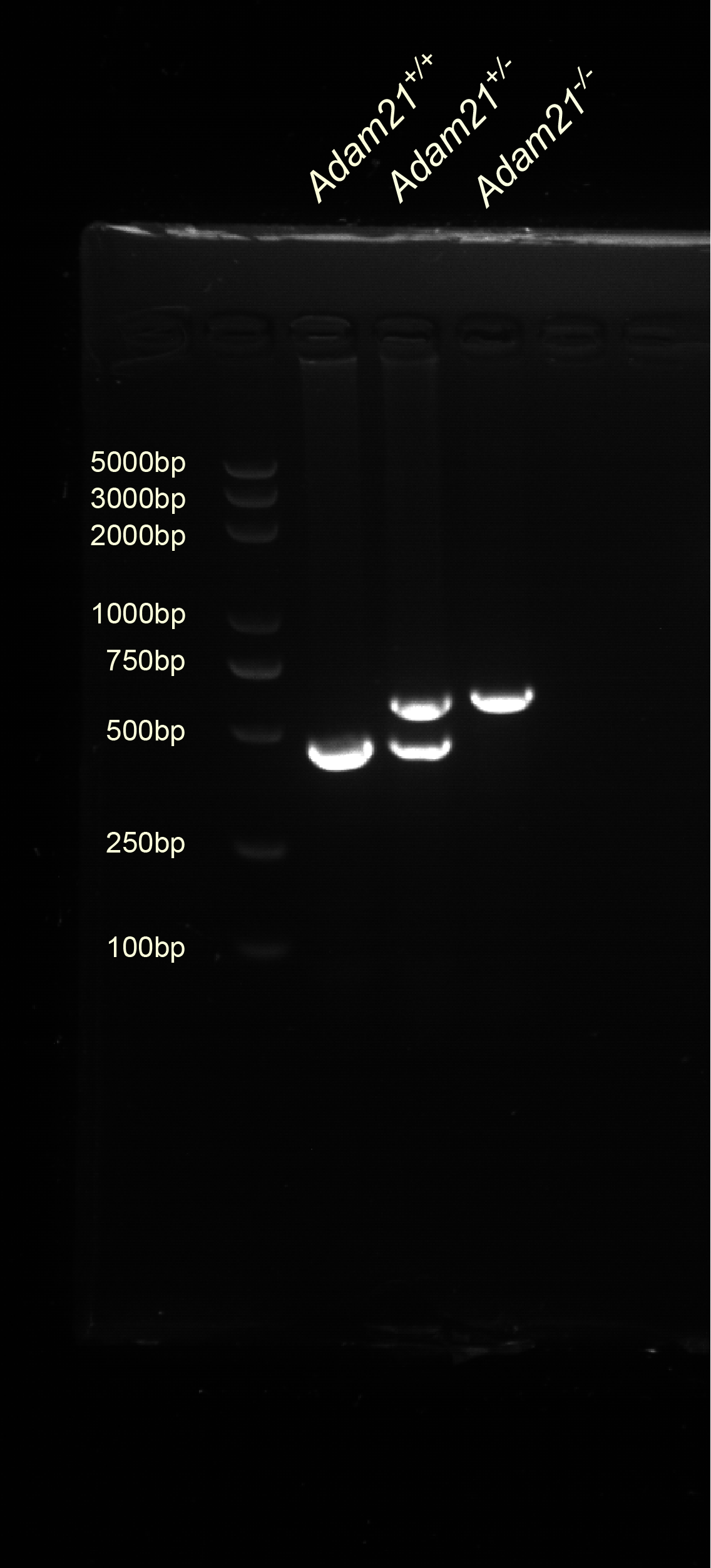

Supplement: Supplemental Information 9 [file peerj-09-12210-s009.zip › Full-length uncropped blots of Adam21 genotyping results--Figure 1/Full-length uncropped blots of Adam21 genotyping results--Figure 1.bmp]

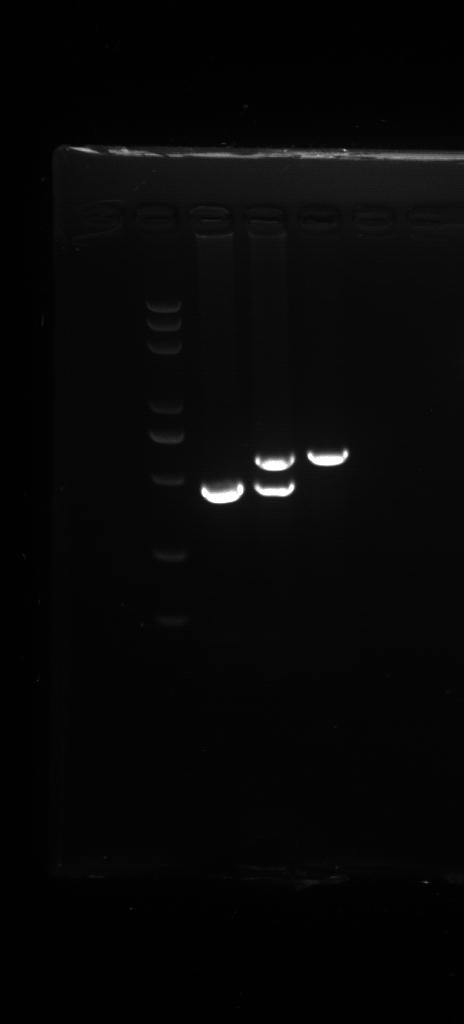

Supplement: Supplemental Information 9 [file peerj-09-12210-s009.zip › Full-length uncropped blots of Adam21 genotyping results--Figure 1/full-length uncropped blots of Adam21 genotyping results-Figure 1.bmp]
